# Urinary concentrations of phthalate metabolites in early pregnancy associated with clinical pregnancy loss in Chinese women

**DOI:** 10.1038/s41598-017-06450-2

**Published:** 2017-07-28

**Authors:** Hui Gao, Yun-wei Zhang, Kun Huang, Shuang-qin Yan, Lei-jing Mao, Xing Ge, Ye-qing Xu, Yuan-yuan Xu, Jie Sheng, Zhong-xiu Jin, Peng Zhu, Xu-guang Tao, Jia-hu Hao, Fang-biao Tao

**Affiliations:** 1 Department of Maternal, Child and Adolescent Health, School of Public Health, Anhui Medical University, Hefei, Anhui China; 2 Ma’anshan Maternal and Child Health (MCH) Center, Ma’anshan, Anhui, China; 3 Anhui Provincial Key Laboratory of Population Health and Aristogenics, Hefei, Anhui China; 4 Johns Hopkins Medical Institution, Baltimore, USA

## Abstract

Limited evidence revealed conflicting results on relationship between phthalate exposure and clinical pregnancy loss (gestational weeks >6). A prospective cohort study in Chinese pregnant women (n = 3220) was conducted to investigate the association between urinary phthalate metabolites and clinical pregnancy loss (gestational weeks 6 to 27; n = 109). Morning urine samples during gestational weeks 5 to 14 (mean 10.42) were collected to measure monomethyl phthalate (MMP), monoethyl phthalate (MEP), monobutyl phthalate (MBP), monobenzyl phthalate (MBzP), mono (2-ethylhexyl) phthalate (MEHP), mono (2-ethyl-5-oxohexyl) phthalate (MEOHP) and mono (2-ethyl-5-hydroxyhexyl) phthalate (MEHHP). The concentrations of low- and high-molecular weight phthalate metabolites (ΣLMWP <250 Da and ΣHMWP >250 Da) were calculated. Adjusted logistic regression models showed increased risks of clinical pregnancy loss in women with higher creatinine- normalized concentrations of MEP, MBP, MEOHP, MEHHP, ΣLMWP and ΣHMWP. Stratified analysis by gestational weeks (10 weeks) of miscarriage indicated positive associations of MEP, MEOHP, MEHHP and ΣHMWP with embryonic loss (during gestational weeks 6 to 10). The only association of foetal loss (during gestational weeks 11 to 27) was observed with MEHHP. Our findings suggested that Chinese women who were exposed to phthalates during early pregnancy had an increased risk of clinical pregnancy loss, especially embryonic loss.

## Introduction

Phthalates are produced on a large scale and extensively used worldwide, causing ubiquitous contamination of the environment^1–4^. General population is continually exposed to phthalates in daily life^5^. Humans are exposed to phthalates through ingestion, inhalation, dermal absorption, and medical treatment^6–9^. These chemicals are quickly metabolized into corresponding monoesters and oxidized monoesters, and are excreted in urine^10, 11^. Urine is the matrix of choice for biomonitoring nonpersistent chemicals including phthalates^12^. The detection rate of phthalates in urine samples collected from pregnant women is nearly 100%, reported by international studies^13–18^. Some phthalates even penetrate placenta^19^ and can be detected in placental tissue^19^, meconium^20^, amniotic fluid^20–22^ and cord blood^23^. These findings suggest that human exposure to phthalates may begin in utero^22^. Therefore, unborn foetuses of pregnant women are regarded as a high-risk group for the potential adverse health effects of phthalate exposure^24^.

In China, clinical pregnancy loss is defined as a miscarriage that a clinically-recognized pregnancy (gestational weeks >6) involuntarily ends before 28 complete weeks^25^. The incidences were reported 6–14% in China, while over 20% in other countries^26^. A recent Chinese study revealed that the incidence of pregnancy loss was 9.04% in rural and 3.75% in urban areas^27^. Pregnancy loss is one of the most common adverse pregnancy outcomes worldwide and causes physical and psychological traumas for pregnant women and their families. Besides, economic burden is heavy for patients themselves and their countries. Pregnancy loss is a multi-factorial disorder linked to interactions between genetic and environmental factors, such as maternal age, smoking, caffeine and alcohol consumption, and exposure to environmental pollutants^28^. The variety of environmental exposures linked to miscarriage continue to increase. Much attention focuses on the environmental endocrine disruptors and phthalates are among them.

Phthalates can interfere with the development of multiple body systems, including reproductive system. Epidemiological evidences show that phthalate exposure may cause adverse pregnancy consequences (e.g. increased blood pressure and decreased blood glucose) and abnormal foetal growth (e.g. low birth weight, preterm birth, decreased gestational age and birth length)^20, 22, 29–32^. To our knowledge, the effect of phthalate exposure on pregnancy loss is still unclear due to insufficient epidemiological studies. Three cohort studies based on small sample sizes showed inconsistent associations between phthalate exposure and pregnancy loss in two countries^33–35^. A cohort study of 128 Danish women found that increased level of mono (2-ethylhexyl) phthalate (MEHP) was associated with higher risk of subclinical embryonic loss (within 6 gestational weeks), and lower risk of clinical pregnancy loss (conception lasting >6 weeks)^33^. A cohort study of 303 American women undergoing medical assisted reproduction revealed significant relationships between di (2-ethylhexyl) phthalate (DEHP) metabolites and biochemical and total pregnancy loss^34^. However, another cohort study of 221 American women had opposite findings that mono (2-ethyl-5-hydroxyhexyl) phthalate (MEOHP) and DEHP were related to decreased risk of subclinical embryonic loss, and insignificant relationship between any analyzed compounds and clinical pregnancy loss^35^. In China, all current conclusions on associations between phthalates and miscarriage are solely based on case-control studies^36–38^. A case-control study (132 clinical pregnancy loss cases and 172 healthy controls) revealed that an increased risk of clinical pregnancy loss was associated with monoethyl phthalate (MEP) and mono-*n*-butyl phthalate (MBP)^36^. Another study suggested that exposure to monoethyl phthalate (MMP) and metabolites of DEHP was associated with missed miscarriage^37^. Peng *et al*.^38^ found that mono-*iso*-butyl phthalate (MiBP) and MBP concentrations in women with unexplained recurrent spontaneous abortion were remarkably higher than those in control group. Differences in study design, population characteristics, sample size, and miscarriage type are likely to contribute to the conflicting results. Therefore, prospective cohort studies are urgently needed to further identify the associations between phthalates and miscarriage in China.

This prospective cohort study based on large sample size was conducted in China. The aim was to investigate the association between phthalate exposure during early pregnancy and clinical pregnancy loss.

## Results

### Demographic characteristics

Demographic characteristics of women who were included (n = 3 220) and excluded (n = 254) in the present study were shown in Supplementary material, Table S1. The BMI was 20.88 ± 2.85 in the enrolled group, significantly lower than that (21.54 ± 3.25) in the excluded group. Besides, the educational level was lower (middle school or below; 19.60% vs. 34.65%) and the proportion of alcohol-drinking was higher (8.32% vs. 3.15%) in the included participants. No significant differences were found between the included and excluded individuals in terms of age, smoking and parity. The detailed information for study participants was summarized in Table 1. Compared to women with single live births, there were significant differences of maternal age, parity and gestational weeks of urine collection in women with clinical pregnancy loss, but no difference was observed regarding prepregnancy BMI, smoking, drinking and education. In terms of stratification descriptions, embryonic loss was significantly associated with gestational weeks of urine collection, while foetal loss was significantly associated with maternal age, prepregnancy BMI, parity and gestational weeks of urine collection.

**Table 1:** Characteristics of participants enrolled in the present study (n = 3,220).

Variable	Clinical pregnancy loss (n = 109)		Single live births (n = 3111)
	Embryonic loss (gestational weeks 6 to 10; n = 64)	Foetal loss (gestational weeks 11 to 27; n = 45)	
Mean ± standard deviation			
Maternal age, y	26.94 ± 4.45	27.29 ± 3.76	26.12 ± 3.62
Pre-pregnancy BMI, kg/m^2^	20.89 ± 2.63	22.10 ± 3.52	20.87 ± 2.84
Current smoking		Number of subjects (%)	
Yes	0(0.00)	0(0.00)	6(0.19)
No	64(100.00)	45(100.00)	3105(99.81)
Alcohol consumption			
Yes	9(14.06)	2 (4.44)	257(8.26)
No	55(85.94)	43(95.56)	2854(91.74)
Educational level			
Middle school or below	12(18.75)	15(33.33)	604(19.41)
High school	15(23.44)	11(24.44)	704(22.63)
Junior college	16(25.00)	10(22.22)	969(31.15)
University or above	21(32.81)	9(20.00)	834(26.81)
Parity			
0	52(81.25)	33(73.33)	2761(88.75)
≥1	12(18.75)	12(26.67)	350(11.25)
Gestational weeks of urine collection	9.61 ± 1.95	9.75 ± 2.07	10.40 ± 2.06

Abbreviation: BMI, body mass index.

### Phthalate metabolite concentrations

The overall creatinine-normalized concentrations of phthalate metabolites were identical to our previous study^39^, as participants were from the MABC. The detectable frequencies of most phthalate metabolites were over 99%, except for MBzP (64%).

### Phthalates and clinical pregnancy loss

The results of logistic regression analyses were shown in Table 2. After adjustments for maternal age, education, parity and gestational weeks of urine collection, concentrations of MEP, MBP, MEOHP, MEHHP, ΣLMWP, and ΣHMWP were significantly associated with an increased risk of clinical pregnancy loss.

**Table 2:** Logistic regression analyses of associations between creatinine-adjusted concentrations of phthalate metabolites and clinical pregnancy loss.

Phthalate	T_1_ (n = 1073)	T_2_ (n = 1074)		T_3_ (n = 1073)	
		No. of loss (%)	OR (95%CI)	No. of loss (%)	OR (95%CI)
MMP	42 (3.91)	31 (2.89)	0.73 (0.45,1.17)	36 (3.36)	0.85 (0.54,1.35)
MEP	26 (2.42)	43 (4.00)	1.73 (1.05,2.84)	40 (3.73)	1.62 (0.98,2.69)
MBP	29 (2.70)	34 (3.17)	1.19 (0.72,1.97)	46 (4.29)	1.59 (1.00,2.56)
MBzP	35 (3.26)	40 (3.72)	1.18 (0.74,1.89)	34 (3.17)	1.00 (0.61,1.61)
MEHP	34 (3.17)	35 (3.26)	1.04 (0.64,1.69)	40 (3.73)	1.20 (0.75,1.92)
MEOHP	28 (2.61)	33 (3.07)	1.24 (0.74,2.07)	48 (4.47)	1.70 (1.05,2.74)
MEHHP	22 (2.05)	38 (3.54)	1.77 (1.03,3.01)	49 (4.57)	2.26 (1.35,3.78)
ΣLMWP	28 (2.61)	37 (3.45)	1.35 (0.82,2.22)	44 (4.10)	1.60 (1.00,2.60)
ΣHMWP	28 (2.61)	32 (2.98)	1.16 (0.69,1.94)	49 (4.57)	1.74 (1.08,2.80)

Abbreviations: OR, odds ratio; CI, confidence interval; T_1/2/3_, the first/second/third tertile of phthalate concentrations. Adjusted for maternal age, education, parity and gestational weeks of urine collection.

### Stratification analyses

Stratification analyses (Table 3) revealed that increased risk of embryonic loss was associated to higher concentrations of MEP, MEOHP, MEHHP and ΣHMWP. For the risk of foetal loss, the only significant association observed was with the higher level of MEHHP.

**Table 3:** Logistic regression analyses of associations of phthalate metabolites with embryonic and foetal loss.

Phthalate	T_1_ (n = 1073)	T_2_ (n = 1073)		T_3_ (n = 1073)	
		No. of loss (%)	OR (95%CI)	No. of loss (%)	OR (95%CI)
Embryonic loss (6 to 10 gestational weeks)^a^					
MMP	22 (2.08)	20 (1.89)	0.91 (0.49,1.68)	22 (2.08)	0.98 (0.54,1.79)
MEP	14 (1.32)	27 (2.55)	1.99 (1.03,3.80)	23 (2.17)	1.65 (0.84,3.23)
MBP	18 (1.70)	17 (1.61)	0.96 (0.49,1.88)	29 (2.74)	1.68 (0.92,3.04)
MBzP	17 (1.61)	27 (2.55)	1.61 (0.87,2.98)	20 (1.89)	1.17 (0.61,2.25)
MEHP	18 (1.70)	22 (2.08)	1.23 (0.66,2.31)	24 (2.27)	1.41 (0.76,2.62)
MEOHP	14 (1.32)	21 (1.98)	1.56 (0.79,3.09)	29 (2.74)	2.16 (1.13,4.11)
MEHHP	13 (1.23)	24 (2.27)	1.88 (0.95,3.72)	27 (2.55)	2.19 (1.12,4.28)
ΣLMWP	17 (1.61)	22 (2.08)	1.32 (0.70,2.50)	25 (2.36)	1.53 (0.82,2.86)
ΣHMWP	14 (1.32)	21 (1.98)	1.53 (0.77,3.02)	29 (2.74)	2.19 (1.15,4.17)
Foetal loss (11 to 27 gestational weeks)^b^					
MMP	20 (1.90)	11 (1.50)	0.53 (0.25,1.11)	14 (1.33)	0.70 (0.35,1.41)
MEP	12 (1.14)	16 (1.52)	1.38 (0.65,2.94)	17 (1.62)	1.55 (0.73,3.29)
MBP	11 (1.05)	17 (1.62)	1.55 (0.72,3.34)	17 (1.62)	1.47 (0.68,3.16)
MBzP	18 (1.71)	13 (1.24)	0.75 (0.36,1.54)	14 (1.33)	0.81 (0.40,1.65)
MEHP	16 (1.52)	13 (1.24)	0.86 (0.41,1.81)	16 (1.52)	1.04 (0.51,2.11)
MEOHP	14 (1.33)	12 (1.14)	0.92 (0.42,2.01)	19 (1.81)	1.28 (0.63,2.58)
MEHHP	9 (0.86)	14 (1.33)	1.69 (0.72,3.94)	22 (2.09)	2.41 (1.10,5.30)
ΣLMWP	11 (1.05)	15 (1.43)	1.36 (0.62,2.98)	19 (1.81)	1.67 (0.79,3.55)
ΣHMWP	14 (1.33)	11 (1.05)	0.81 (0.36,1.80)	20 (1.90)	1.36 (0.68,2.73)

Abbreviations: OR, odds ratio; CI, confidence interval; T_1/2/3_, the first/second/third tertile of phthalate concentrations. ^a^Adjusted for education and gestational weeks of urine collection. ^b^Adjusted for age, prepregnancy BMI, education, parity and gestational weeks of urine collection.

## Discussion

In the present study, we observed that both ΣLMWP (including MEP and MBP) and ΣHMWP (MEOHP and MEHHP) during early pregnancy were associated with clinical pregnancy loss, especially with embryonic loss, among Chinese women in the study. According to the results of previous related studies^33–38^, the magnitude of associations and types of phthalate metabolites with pregnancy loss remain inconsistent. We hypothesized that the discrepancy between the present study and the previous studies was attributed partially to the differences in maternal characteristics, study design, sample size and miscarriage type and partially to co-linearity among phthalate metabolites. For instance, previous studies were from three different countries (Denmark, America and China) and both cohort^33–35^ and case control study designs^36–38^ were employed. All studies had small sample sizes which would create large variations among studies. For example, cohort studies conducted by Toft *et al*.^33^ and Jukic *et al*.^35^ included only 128 and 221 pregnant women, while Messerlian *et al*.^34^ recruited 303 pregnant women undergoing medically assisted reproduction. Among case-conrol studies, Mu *et al*.^36^ enrolled 132 clinical pregnancy loss cases and 172 healthy controls; Yi *et al*.^37^ included 150 women with missed miscarriages and 150 healthy controls; Peng *et al*.^38^ enrolled only a total of 30 cases with unexplained recurrent spontaneous abortion and 30 controls. In addition, these three case-control studies recruited individuals with different miscarriage types. As indicated above, phthalate metabolites are usually co-existing agents thus highly correlated with each other. Those were found significant in the analyses could be just surrogates for other co-existing ones. This was why most studies indicated that exposure to this family of compounds during pregnancy increased the risks of miscarriage, although which members were related to adverse pregnancy outcomes is still debatable.

The unfavorable effect of phthalates in population is similar to observations from experimental studies. Evidences from rat and mouse models showed chemicals such as DEHP, di-*n*-butyl phthalate (DBP), diisobutyl phthalate, and diethyl phthalate induced decreased numbers and sizes of litters, reduced live-born offspring or embryo survival, and increased midpregnancy abortions, postimplantation loss, and intrauterine absorption^40–47^.

Our results indicated embryonic loss was more sensitive to phthalate exposure. The Danish Prospective Cohort Study of Fecundity (1992–1994)^33^ found that a relationship between MEHP and subclinical pregnancy loss [adjusted odds ratio (OR_adj_) = 10.83 and 40.67, 95% confidence interval (95%CI) = 1.16−101.48 and 4.48−369.50 for the second tertile (T_2_) and T_3_, respectively, compared with T_1_), whereas an inverse association between MEHP and clinical pregnancy loss was unexpectedly identified (OR_adj_ = 0.17, 95%CI = 0.03−0.95 for T_2_ compared with T_1_). The Environment and Reproductive Health Study (2004–2014)^34^ suggested that DEHP metabolites were associated with both biochemical and total pregnancy loss. Therefore, it was suggested that the pregnancy loss was more susceptible to phthalate exposure when the gestational age was shorter. However, the North Carolina Early Pregnancy Study (1982–1986)^35^ showed that exposure to MEOHP and DEHP reduced the risk of subclinical embryonic loss (*p* = 0.04 and 0.001, respectively; ORs and 95%CIs were not shown in the article), but none of the analyzed compounds were related to clinical pregnancy loss. Additional human studies are warranted to verify these associations.

Phthalates are known as environmental endocrine disrupters. Some phthalates [i.e., DEHP, DBP, butyl benzyl phthalate (BBzP), and their metabolites] reportedly reduced the production of oestradiol and progesterone in the ovaries through a receptor-mediated signalling pathway (cAMP and peroxisome proliferator-activated receptors)^46, 48, 49^. Their adverse effects on endocrine functioning is likely to change the circulating levels of hormones responsible for maintaining pregnancy. In addition, phthalates were found associated with increments of inflammation biomarkers (i.e., IL-6 and IL-10) and oxidative stress (i.e., 8-hydroxyguanosine and 8-isoprostane)^13, 50, 51^. Some investigations suggested pregnancy failure might also be mediated by the recruitment of a highly activated uterine natural killer cell^52^. Stress-triggered immune and endocrine events during pregnancy could induce miscarriage^52^. Another hypothesis suggested that angiogenic factors (placental growth factor and soluble fms-like tyrosine kinase-1) and placenta log interspersed nuclear elements-1 methylation were decreased by phthalates^29, 53^. Therefore, phthalates could disrupt placental development and function during gestation. This is particularly concerning, because the placenta establishes close contact between the mother and foetus and enables the exchange of gases, nutrients, and waste products^54, 55^. Notably, Mose *et al*.^19^ determined that short-chained phthalate monoesters could cross the placenta by slow transfer, but long-chained monoesters exhibited no placental transfer. These evidences biologically support the hypothesis that some phthalates could have adverse effects on the clinical pregnancy loss.

To our knowledge, this study is the first prospective cohort study in China that has investigated the association of phthalate exposure with clinical pregnancy loss, embryonic loss, and foetal loss. However, there are some limitations that should be addressed. Firstly, the proportion of pregnancy losses in the present study is substantially low. We hypothesized several explanations. One is that miscarriage occurred prior to clinical detection or first prenatal visits. Another is that our participants had higher socioeconomic status (SES), since miscarriage risk was increased with lower SES status^27^. As the MABC is a hospital-based study, the third possible explanation is that the community derived data were not taken into account in this study, thereby causing a lower incidence. In this situation, it was likely that we underestimated the relationship of miscarriage with phthalate metabolites. Secondly, we only used a single-spot urine sample for assessing the phthalate exposure in early pregnancy. It has been reported that a single measurement could be used as a typical measurement for a period ranging from weeks to months, when sources or exposure patterns are consistent^17^. Therefore, it is unlikely to affect our results. Finally, we did not differentiate the two isomers of MnBP and MiBP; they were combined and designated as ‘MBP’ in our study.

In conclusion, exposures to MEP, MBP, MEOHP, MEEHP, ΣLMWP and ΣHMWP in early pregnancy (5 to 14 weeks’ gestation) are independent risk factors for clinical pregnancy loss, especially for embryonic loss, in Chinese pregnant women. These findings should be verified by additional scientific evidences.

## Methods

### Study population

The present study was conducted on the basis of the Ma’anshan Birth Cohort (MABC) study. From May 2013 to September 2014, the MABC consecutively recruited 3,474 pregnant women at their first visits (gestational weeks 5 to 14) in Ma’anshan Maternal and Child Health Centre in China^56^. Meanwhile, questionnaires regarding demographic characteristics were obtained and first-morning urine samples were collected for measuring phthalate metabolites. Information on pregnancy/birth outcomes, such as single live birth, clinical pregnancy loss, and stillbirth, was obtained from medical records.

Of the 3,474 women, 81 women with ectopic pregnancy (n = 2), stillbirth (n = 10), therapeutic abortion (n = 30), or multiple gestations (n = 39) were excluded. 173 women (162 women with live births and 11 women with miscarriages) whose urine specimens were unavailable were also excluded from the analysis. The remaining 3,220 women, consisting of 3,111 women with single live birth and 109 women with clinical pregnancy loss, were included in the current study. Of the 109 women, 64 women underwent embryonic loss (gestational weeks 6 to 10) and 45 women underwent foetal loss (gestational weeks 11 to 27)^57^.

All the participants provided written informed consents. The recruitment and subsequent follow-up protocols (including the urinary analysis of phthalates) were both approved by the ethics committee of Anhui Medical University (approval No. 2013119). All the study methods were performed in accordance with the relevant guidelines.

### Covariates

A series of potential confounding covariates were selected, including maternal age (continuous), prepregnancy body mass index (BMI; continuous), current cigarette consumption (yes or no), alcohol consumption (yes or no), educational level (middle school or below, high school, junior college, university or above) and parity (0 or >1).

### Exposure assessment

Seven urinary phthalate metabolites including MMP, MEP, MBP, monobenzyl phthalate (MBzP), MEHP, MEOHP, and mono (2-ethyl-5-hydroxyhexyl) phthalate (MEHHP) were measured through high-performance liquid chromatography-tandem mass spectrometry (6410LC-MS, Agilent Technologies Co., Santa Clara, CA, USA), using a previously reported methodology with modifications^58, 59^. Concentrations below the limit of detection (LOD) were assigned with LOD/√2 for calculations. Urine creatinine was measured using a creatinine assay kit based on the picric kinetic method (Jiancheng Bioengineering Institute, Nanjing, China).

### Statistical analyses

Phthalate metabolites were categorized into the low- (ΣLMWP; <250 Da) and high-molecular weight phthalates (ΣHMWP; >250 Da)^60^, which were calculated according to the following equations:

$$LMWPs\,({\rm{\mu }}\mathrm{mol}/{\rm{L}})=\frac{MMP({\rm{\mu }}{\rm{g}}/{\rm{L}})}{M{W}_{MMP}({\rm{g}}/{\rm{mol}})}+\frac{MEP({\rm{\mu }}{\rm{g}}/{\rm{L}})}{M{W}_{MEP}({\rm{g}}/{\rm{mol}})}+\frac{MBP({\rm{\mu }}{\rm{g}}/{\rm{L}})}{M{W}_{MBP}({\rm{g}}/{\rm{mol}})}$$

$$\begin{array}{rcl}HMWPs({\rm{\mu }}\mathrm{mol}/{\rm{L}}) & = & \frac{MBzP({\rm{\mu }}{\rm{g}}/{\rm{L}})}{M{W}_{MBzP}({\rm{g}}/{\rm{mol}})}+\frac{MEHP({\rm{\mu }}{\rm{g}}/{\rm{L}})}{M{W}_{MEHP}({\rm{g}}/{\rm{mol}})}\\  &  & +\,\frac{MEOHP({\rm{\mu }}{\rm{g}}/{\rm{L}})}{M{W}_{MEOHP}({\rm{g}}/{\rm{mol}})}+\frac{MEHHP({\rm{\mu }}{\rm{g}}/{\rm{L}})}{M{W}_{MEHHP}({\rm{g}}/{\rm{mol}})}\end{array}$$

The phthalate metabolite concentrations were creatinine-normalized to account for the impact of maternal urine dilution.

The creatinine-normalized concentrations of phthalate metabolites had a skewed distribution. Therefore, phthalate metabolite concentrations were natural log transformed. Women were grouped into tertiles according to the concentrations (T_1_ < *P*_1/3_, *P*_*1/3*_ < T_2_ < *P*_*2/3*_ and T_3_ > *P*_*2/3*_). Logistic regressions were used to analyze the odds ratios (ORs) and 95% confidence intervals (95% CIs) for the risks of clinical pregnancy loss. Each phthalate was included in one model, because they were all correlated with each other with Spearman *r* ranging from 0.04 to 0.61 (all *p* values < 0.05). Covariates which were significantly associated with clinical pregnancy loss, embryonic loss and foetal loss (*p* < 0.05) were adjusted for corresponding models. Education level is reportedly played an important role in pregnancy loss risk, therefore it was included as a confounder^27^. Stratification analyses were also performed by types of miscarriage.

All statistical analyses were performed using the SPSS software (Version 10.0), and *p* < 0.05 was considered statistically significant.

## Electronic supplementary material

Supplementary Materials
