# Supplementary material for: Urinary concentrations of phthalate metabolites in early pregnancy associated with clinical pregnancy loss in Chinese women
Source: Sci Rep. 2017 Jul 28;7:6800. doi: 10.1038/s41598-017-06450-2 (PMC5533765; doi:10.1038/s41598-017-06450-2)
Supplement: Supplementary file 1 — Supplementary Materials [file 41598_2017_6450_MOESM1_ESM.pdf]

Supplementary material

Urinary concentrations of phthalate metabolites in early pregnancy associated with clinical pregnancy loss in Chinese women

Hui Gao [a], Yun-wei Zhang [a], Kun Huang [a], Shuang-qin Yan [b], Lei-jing Mao [a], Xing Ge [a], Ye-qing Xu [b], Yuan-yuan Xu [a, c], Jie Sheng [c], Zhong-xiu Jin [c], Peng Zhu [a, c], Xuguang Tao [d], Jia-hu Hao [a, c] & Fang-biao Tao [a, c *]

Table S1. Comparisons of the characteristics of women who were included (n = 3220) and excluded (n = 254) in the present study.

Variable	Women enrolled (n = 3220)	Women excluded (n = 254)	*p-value*
Maternal age, y	26.15±3.65	26.44±4.21	0.28
Pre-pregnancy BMI, kg/m^2	20.88±2.85	21.54±3.25	<0.01
Current smoking			0.41
Yes	6(0.19)	1(0.39)	
No	3214(99.81)	253(99.61)	
Alcohol consumption			<0.01
Yes	268(8.32)	8(3.15)	
No	2952(91.68)	246(96.85)	
Educational level			<0.01
Middle school or below	631(19.60)	88(34.65)	
High school	730(22.67)	56(22.05)	
Junior college	995(30.90)	64(25.20)	
University or above	864(26.83)	46(18.11)	
Parity			0.16
0	2846(88.39)	217(85.43)	
≥ 1	374(11.61)	37(14.57)	
Clinical pregnancy loss			
Yes	109(3.39)	11(4.33)	<0.001
No	311196.61)	243(95.67)	

Abbreviation: BMI, body mass index.
